# Book Reviews

**Published:** 1831-08

**Authors:** 


### CRITICAL ANALYSES.

Quæ laudanda forent, et quæ culpanda, vicissim

Illa, prius, cretâ; mox hæc, carbone, *notamus*.—PERSIUS.

*Essays and Orations, read and delivered at the Royal College of Physicians; to which is added, an Account of the Opening of the Tomb of King Charles I.* By Sir HENRY HALFORD, Bart. M.D., G.C.H., President of the College.—8vo. pp. 192. Murray, London.

BY all who love to blend the amenities of science with the severer studies which medicine and surgery demand, and thus by literature to lighten the labours, or sooth the anxieties with which the practical duties of an arduous profession must abound, these "*deliciæ*" of Sir HENRY HALFORD will be welcomed as beautiful oases in what, without some such philosophic relaxations, might too truly be regarded as the unvaried routine of a dull professional career. Were he otherwise utterly unknown, these Essays and Orations would stamp their author with celebrity, as a man of no common parts, as an admirable critic, as the possessor of an elegant and highly cultivated mind: but, standing as he does at the summit of his profession, they can add little to the fame, and nothing to the fortune which he already so deservedly enjoys: still they form pleasing samples of the mode in which the leisure of a physician, who is also a philosopher, may be passed, and should moreover be received as proofs that the most extensive medical knowledge is not (as sometimes thought) incompatible with the highest classical attainments.

Here, in the space of less than two hundred, not closely printed, octavo pages, we have presented to our notice six essays, two orations, and an account of the appearances observed on the opening the coffin of King Charles I. at Windsor, with some illustrative extracts from "Clarendon's History of the Rebellion," and "Wood's Athenæ Oxonienses," (the two latter forming an appendix:) so it will be evident that none of the productions can be tedious from prolixity, and (whether our readers concur in all Sir Henry's opinions, or, like ourselves, occasionally dissent,) we think we can venture to promise they will not be found irksome from any other cause.

Of two of the dissertations, viz. the paper read at the College of Physicians on SHAKSPEARE's test of Madness, and that on the Καυσος of ARETÆUS, we have already made mention in our Journal: to them, therefore, we shall not now particularly advert, but confine our present observations, more especially as they are the most strictly medical, to the two or three with which the volume opens.

The first essay, on the *Climacteric Disease*, contains some curious matter for reflection, but it is scarcely so definite in its terms as, medically considered, we could have wished: it commences with the truism, that

"The human constitution, in its progress to maturity, undergoes repeated changes, by which its energies are developed; and it reaches at length that degree of perfection, whatever it may be, of which the individual nature is capable." (P. 3.)

It then proceeds,

"Other changes, too, of an important kind, generally occur in the decline of life; and philosophers have amused themselves with calculating the period at which these must happen, from the successive alterations which the frame underwent in early youth; not taking into their account the influence which moral causes have in our progress through life, in disturbing the regularity of natural processes, nor considering that various accidents and habits of living more frequently determine the number of a man's years, than the strength of the stamina with which he was born." (P. 3.)

The averment that "the influence of moral causes" in affecting health and shortening life, are not by philosophers taken into account, we scarcely can esteem correct; for nothing is more common than for both the wise and simple to observe, of debauchees, as well as of persons employed in certain arts and trades, that "they are old before their time," or that "they are more aged in constitution than in years," or that a short life is frequently the consequence of a merry one. It seems, then, on the contrary to us, that no doctrine is more generally admitted than that which declares the influence which moral causes have in determining the period of man's sojourn here: men may be, nay constantly are, worn out, like horses, not only by years, but by excess of any kind, whether of labour, of study, of care, of pleasure (falsely so called), of abstinence from, or of indulgence in, sensual gratifications; all which prematurely kill, and thus prevent men dying a truly natural death: or, to state the question in other words, "that various accidents and habits of living more frequently determine the number of a man's years, than the strength of the stamina with which he was born."

Sir Henry thus proceeds:

"It will not be disputed, however, that the alteration of the condition of the system in age is not so well marked as that which took place in the beginning of life; and it must be admitted, that in some persons who have reached very great age, no such alteration has been manifested at the epochs which have been called climacteric. The period of the occurrence of this change in men, in general, is so very irregular, that it may be occasionally remarked at any time between fifty and seventy-five years of age; and I will venture to question, whether it be not, in truth, a disease, rather than a mere declension of strength and decay of the natural powers. To the argument by which it is maintained that it is mere decay, it may be sufficient to answer, that men frequently rally from the languid and feeble condition of their system into which this change had thrown them; become, to a certain degree, themselves again, and live for years afterwards.

"But it appears to me to have the signs of a marked and particular disease; and I would describe it as a falling away of the flesh in the decline of life, without any obvious source of exhaustion, accompanied with a quicker pulse than natural, and an extraordinary alteration in the expression of the countenance.

"Sometimes the disorder comes on so gradually and insensibly, that the patient is hardly aware of its commencement. He perceives that he is sooner tired than usual, and that he is thinner than he was; but yet he has nothing material to complain of. In process of time, his appetite becomes seriously impaired; his nights are sleepless; or, if he gets sleep, he is not refreshed by it. His face becomes visibly extenuated, or perhaps acquires a bloated look. His tongue is white, and he suspects that he has a fever.

"If he ask advice, his pulse is found quicker than it should be, and he acknowledges that he has felt pains occasionally in his head and chest, and that his legs are disposed to swell; yet there is no deficiency in the quantity of his urine, nor any other sensible failure in the action of the abdominal viscera, excepting that the bowels are more sluggish than they used to be.

"Sometimes the headach is accompanied with vertigo; and sometimes severe rheumatic pains, as the patient believes them to be, are felt in various parts of the body, and in the limbs; but, on inquiry, these have not the ordinary seat, nor the common accompaniments of rheumatism, and seem rather to take the course of nerves, than of the muscular fibres.

"In the latter stages of this disease, the stomach seems to lose all its powers; the frame becomes more and more emaciated; the cellular membrane, in the lower limbs, is laden with fluid; there is an insurmountable restlessness by day, and a total want of sleep at night; the mind grows torpid and indifferent to what formerly interested it; and the patient sinks at last, seeming rather to cease to live, than to die of a mortal distemper.

"Such is the ordinary course of this disorder in its most simple form, when it proves fatal. When the powers of the constitution are superior to the influence of the malady, the patient loses his symptoms gradually, recovers his rest and his appetite, and, to a certain degree, his muscular strength and flesh; but the energies of his frame are never again what they were before, nor does the countenance recover its former volume and expression." (P. 4.)

The foregoing extracts, though elegantly written, if considered as the diagnosis of a "distinct and particular distemper," are certainly deficient in perspicuity: neither do we find that the context relieves the indeterminateness of which we have, and we think not without some shew of reason, ventured to complain: for if, as appears to us to be the case, the learned author intends to describe a "marked and particular disease," such as tic douloureux, the Καυσος of Aretæus, or any of those maladies, either of mind or body, referred to in the subsequent papers, what are the symptoms by which the Morbus climactericus is to be distinguished? In the first place, we are told "it must be admitted that, in some persons, who have reached a very great age, no such alteration has been manifested at the epochs which have been called climacteric;" that "the period of the occurrence of this change in men, in general, is so very irregular, that it may occasionally be remarked at any time between fifty and seventy-five years of age," (p. 4:) and again, further on, with regard to women, we are told,

"That though this climacteric disease is sometimes equally remarkable in women as in men, yet most certainly I have not noticed it so frequently nor so well characterized in females. Perhaps the severe affections of their system, which often attend the bearing of children, or, what is more likely, the change which the female constitution undergoes at the cessation of the catamenia, may render subsequent alterations less perceptible." (P. 10.)

Thus, we find that in men its signs are very equivocal, and in women less certain than in men. However, the chief diagnostic symptoms are described to be a "*falling away of the flesh in the decline of life, without any obvious cause of exhaustion, accompanied with a quicker pulse than natural, and an extraordinary alteration in the expression of the countenance.*" But blunted excitability of the digestive apparatus, worn out either by excess of use or years, would appear to us a sufficiently obvious source of exhaustion, especially when we are told that the appetite becomes seriously impaired; that, "in the latter stages of this disease, the stomach seems to lose all its powers," whence it, almost of necessity, would follow that "the frame becomes more and more emaciated, the cellular membrane in the lower limbs laden with fluid," &c. &c.; and the view we take will be more decidedly favored when we notice this further statement:

"But it is seldom that we have an opportunity of observing this malady in its simple form; and never, I believe, but in a patient whose previous life has been entirely healthy. We find it generally complicated with other complaints, assuming their character, and accompanying them in their course; and perhaps this may be the reason why we do not find the climacteric disease described in books of nosology as a distinct and particular distemper. It blends itself with the effects of any fixed organic mischief in the constitution; takes on the appearance of any periodical irritation to which a patient may have been subject, or adopts the features of a casual disease. When it is associated with organic mischief, it is difficult to distinguish the climacteric complaint from that train of symptoms which commonly supervenes, sooner or later, on diseased structure; but its presence ought to be suspected if the complaints are all unusually exasperated, if a fatal result be threatened earlier than is usual in the common course of things, and, above all other indications, if that character be impressed on the countenance which peculiarly distinguishes the disorder." (P. 8.)

What "that character," both here and elsewhere mentioned as "an extraordinary alteration in the expression of the countenance," and as *peculiarly distinguishing* this disorder, may be, we are not told; and we are hence unwillingly compelled to turn our attention from *this sign, which is peculiar*, to those general symptoms which are common to many other ailments.

If, in this essay, it had been intended to state that the gradual decay of man's corporeal frame, in which the very act of living is but the pioneer to death, whence the faster (to use a common phrase) a man lives, the sooner he dies; if, we repeat, it had been intended to affirm that this climax might be much hastened, or the epoch become more suddenly and surely marked, as to its climacteric change, by the supervention of any ordinary disease, to which all are liable, we should have perfectly agreed in the justice of the statement; but then we should not have considered such facts as any evidence that the climax is a disease itself: we readily grant that a cold, or any malady, however slight, will more disturb and harass a weakened and half-worn-out constitution, and perhaps even, in such a decaying frame, prove fatal, which, in earlier life, or stronger years, would have been little more than an unregarded ailment; but then we feel compelled to doubt whether it be so philosophic to consider the powers of life, wearing to the close, a climacteric disease, heightening the malevolence of the incidental malady, as to believe that the fortuitous disorder is comparatively more powerful, and assumes its dangerous or fatal form, rather from the waning energy of the *vis naturæ*

*medicatrix,* than that it is aggravated by the coincidence and coexistence of a separate disease, e.g. the climacteric.

When persons in advanced life, or suffering from premature old age, "become sooner tired than usual," perceive themselves "getting thinner," but yet have "nothing material to complain of;" when, "in process of time, the appetite becomes seriously impaired," &c., such changes we should think rather evidences of a gradual wear or decay of nature, than the symptoms of a *marked and particular disease,* using that term in its ordinary acceptation. Neither can we agree that, "to the argument by which it is maintained, that it is mere decay, it may be sufficient to answer, that men frequently *rally* from the languid and feeble condition of their system into which this change had thrown them, and live for years afterwards;" for, in the second following page, it is truly stated, that "when the powers of the constitution are superior to the influence of the malady, the patient losing his symptoms gradually, recovers his rest and appetite, and, to a certain degree, his muscular strength and flesh," still "*the energies of his frame are never again what they were before,*" nor does the countenance recover its former volume and *expression.* Had it been merely a disease affecting certain functions, and not the effect of a gradual impairment of the energies of life, and decay of the general structure, we see no reason why the sufferer should not, occasionally at least, as perfectly recover therefrom as men do from diarrhœa, catarrh, inflammation, and fever, every day; for the falling away is declared to be without any obvious source of exhaustion, and therefore cannot be supposed to be of structural origin: hence we think the doctrine of a gradual decay of the powers of life, and wearing out of the frame which it inhabits, receives much support even from this paper, which intends to controvert it; for we can, on this principle, easily conceive that, when a constitution has been borne down by excessive indulgencies or the weight of years, that it should, after a time, become far less elastic, and at length cease to offer such resistance as previously to accidental disorders, and thus that attacks, once trifling, may, under such circumstances, be serious, or even fatal; and that, when no such diseases supervene, the decay may gradually go on, until the vital organism can no longer perform the actions necessary to life, "and the patient sink at last, seeming rather to cease to live, than to die of a mortal distemper." And thus, if we understand it right, it would, from this most graphic description of such a death, appear that the patient is *not* killed by any "marked or particular disease," but *dies merely because*

*he cannot*, with his worn-out frame, *any longer live:* or, as our author in another place observes, in the classic language of antiquity, "Non tam mori v[illegible]etur quam dulci, et alto sopore excipi."

But what does the writer say "of the various immediate causes to which this malady may [illegible]e its commencement;" or, as we would rather read the sen[illegible]ce, what are the means which hasten this natural decay? Why, that "there is none more frequent than a common cold." "When the body is predisposed to this change, any occasion of feverish excitement, and a privation of rest at the same time, will readily induce it." This is most true of the natural declension which we contend should be considered rather a decay than a disease: we think we could adduce a case immediately in point: we have had under our occasional care, for many years, an old lady, now almost eighty-eight, who has been, for nearly half a century, subject to violent attacks of catarrh, of much more than common severity: formerly she used to recover from them to all appearance perfectly, and fully to regain her strength, but of late years, although the disease has become much less violent in its recurrences, she rallies far less completely from its attacks; her strength gradually diminishes; she evidently gives way more and more to less and less force in the attacks; and on the last recurrence, from which she is now but slowly recovering, the exhaustion was so complete, and the prostration so excessive, that, although the catarrh was little more than a slight June cold, in twelve hours she was unable to lift her cup to her mouth, and for many days (to repeat the author's beautiful expression,) she was hourly expected to "sink, seeming at the point rather of ceasing to live, than of dying of a mortal distemper."

But to proceed with the enumeration of the exciting causes of the climacteric disease, or, as we believe, of the means of hastening the natural decay: Sir Henry says,

"I have known an act of intemperance, where intemperance was not habitual, the first apparent cause of it. A fall, which did not appear of consequence at the moment, and which would not have been so at any other time, has sometimes jarred the frame into this disordered action. A marriage contracted late in life has also afforded the first occasion to this change; but, above all, anxiety of mind and sorrow have laid the surest foundation for the malady, in its least remediable form.

"The effects of grief on the body, physicians have daily occasion to witness and deplore; but they remark, that its influence is very different at an early from what it is at a late period of life. A mind actively engaged, in youth, in the pursuit of fame and fortune, is hardly vulnerable by any disaster which does not immediately stop its career of success; and if a deep impression be made by misfortune, new schemes of ambition and the gradual influence of time contribute to obliterate it; but sorrow late in life has fewer resources, and more easily lets in disease. Have a man's circumstances been suddenly overwhelmed by some unexpected calamity? there is not time to repair his losses, to recover his station in society, and he pines in gloomy despondency. Or has death inflicted the wound in his peace of mind? At this time of life, it may be, the partner of all his happiness and all his care has been torn from him, or a child, who had grown up to be his comfort and support, or perhaps a friend, a contemporary, with his regret for whom there is mixed an apprehension that the next blow may fall on himself; and if at this moment a survey of past life be not more consolatory than the prospect of what remains, adieu to that animating and enlivening hope which is cheerfulness, which is health." (P. 11.)

The above detail has been drawn by a master hand, and comprises facts which a master eye could alone thus accurately perceive: still we must repeat, that to us it seems a graphic detail rather of natural decay and degradation, to which excess or disappointment lend a helping hand, than as containing the symptoms of any marked or particular disease. What is more likely than intemperance to hasten our natural decay? the intemperate can seldom boast of "a green old age, a lusty winter." What more likely than "a marriage contracted late in life" to hasten the weary traveller towards his final goal? in such a case, connubial rites would be any thing but connubial bliss. CULLEN's "Veneris Excessus" (one of his most frequently repeated predisponents,) might here be regarded as truly present, and present not only as an occasional, but a constant exciting cause of debility; hence hastening the catastrophe, by journeying more rapidly through disease to death.

Again, when sorrow, unmitigated by religious hope, merges into despondency; when the bereavement of relations, the sudden loss of friends, the rending of all those ties, the multiplied attachments of which bind the aged, in their offspring, to the earth, and can alone explain their clinging in extremity to life; what more likely than that, if these bands be rudely severed, the sufferer should "bid adieu to that animating and enlivening hope which is cheerfulness, which is health?"

The second essay, "*on the Necessity of Caution in the Estimation of Symptoms in the last Stages of some Diseases,*" will be found a very interesting, and in many respects important and valuable, paper: from it we shall make some copious extracts, and confine our running comments within the narrowest possible bounds.

" It often happens at the latter end of some diseases, both of an acute and a chronic nature, that appearances present themselves of a very equivocal and delusive nature, with which the issue of the malady does not correspond. This is most frequently the case when the resistance of the constitution against the influence of the disease has been long protracted, or when the struggle, though short, has been very violent. Here a pause in nature, as it were, seems to take place; the disease ' has done its worst,' all strong action has ceased, the frame is fatigued by its efforts to sustain itself, and a general tranquillity pervades the whole system. This condition of comparative ease the eager wishes of friends misconstrue into the commencement of recovery, and the more readily so, as the patient himself being appealed to, to confirm their anxious hopes, having lost some of his sufferings, admits, perhaps, that he is better." (P. 21.)

As a caution to the junior part of our profession, Sir Henry adds,

" I have seen the fallacious truce in four or five instances of inflammation of the brain, particularly where the membranes covering it have been inflamed, producing phrenzy.

" A young gentleman of family, about twenty-five years of age, took cold whilst under the influence of mercury. The fever increased daily, until it was accompanied at last by so much fever and delirium as made it necessary to use not only the most powerful medicines, but also personal restraint. At length, after three days of incessant exertion, during which he never slept for an instant, he ceased to rave, and was calm and collected. His perception of external objects became correct, and they no longer distressed him, and he asked, pressingly, if it were possible that he could live? On being answered tenderly, but not in a way calculated to deceive, that it was probable he might not, he dictated most affectionate communications to his friends abroad, recollected some claims upon his purse, ' set his house in order,' and died the following night. The reason why so unfavorable an opinion was entertained of his state, was, that the apparent amendment was not preceded by sleep, and was not accompanied by a slower pulse; two indispensable conditions, on which only a notion of real improvement could be justified. But here was merely a cessation of excitement occasioned by a diminution of power, and by a mitigated influence of the action of the heart upon the brain.

" In inflammation of the bowels, generally, it is so notorious that mortification often follows a cessation of pain, that I do not think it necessary to dwell upon this form of disease with a view of cautioning physicians; but in that partial inflammation of the intestines which a strangulation of a portion of it in hernia produces, how often have I had occasion to deplore the disappointment and broken hopes of relatives, who, having been made happy by the assurance of the surgeon that he had reduced the protruded bowel, and that now all would be well, in only a few hours afterwards were doomed to lament the patient's death! It is an invariable rule with me still to consider life as in jeopardy, until the intestines shall have performed their functions again; all irritation having left the stomach, and the skin remaining universally and equally warm." (P. 23.)

The rule here, with some emphasis, insisted on, as to the prognosis in hernia, previous to the intestines having resumed their natural functions, we have always regarded as the invariable, and still incline to believe the almost invariable rule not only with Sir Henry, but with every well-informed physician and surgeon. We can vouch that, when ourselves were in the schools, it was always so laid down, "ex cathedra," and propounded as the law of the profession that, until the bowels had duly acted, the patient could not be considered safe; and although we have known, and we think with propriety, a surgeon, after the operation for strangulated hernia, congratulate the patient on the reduction of the gut, still we never knew one who had the temerity to assure the friends that all would in consequence be well, and doom them to deplore, in a few hours afterwards, their own broken hopes and their relation's death.

The cases of abscess in the liver simulating ague, are well worthy perusal; as also the caution as to the prognosis in severe cases of variola, and in extensive burns; but for these we must refer to the volume. The observations on dropsy in the chest, although pretty generally familiar, cannot be too often repeated: Sir Henry says, it is a disease

"In attending which a physician should be on his guard when he gives an opinion in the advanced stages of it. We have all seen, in cases of hydrothorax, a most material mitigation of the embarrassment in breathing ensue on the legs swelling; so great a one, indeed, that the patient and his friends have flattered themselves that no ill remained beyond the hydropic enlargement of the lower extremities. I have to remark, that if this swelling of the legs disappear without an increased discharge of urine, the patient generally dies very soon, and very frequently suddenly; whereas, if an ample increased secretion by the kidneys follow the relief of dyspnœa, then every good hope of a temporary recovery, at least, may be fairly entertained; though it should be acknowledged that this species of dropsy, above all the others, is most apt to return." (P. 27.)

But the concluding case is the most instructive of the whole, and goes far to prove an opinion frequently advocated, that the suppression of any secretion, whether of urine or of bile, is much more serious than its retention. The case detailed is one of paralysis of the kidney, a disease of very rare occurrence: Sir Henry says, he has "seen only five instances of it in twenty-seven years; the last was about two years since, and an exact copy of all the others." The details are shortly as follow:

"A very corpulent, robust farmer, of about fifty-five years of age, was seized with a rigor, which induced him to send for his apothecary. He had not made water, it appeared, for twenty-four hours; but there was no pain, no sense of weight in the loins, no distention in any part of the abdomen, and therefore no alarm was taken till the following morning, when it was thought proper to ascertain whether there was any water in the bladder, by the introduction of the catheter, and none was found. I was then called, and another inquiry was made, some few hours afterwards, by one of the most experienced surgeons in London, whether the bladder contained any urine or not, when it appeared clearly that there was none. The patient sat up in bed and conversed as usual, complaining of some nausea, but of nothing material in his own view; and I remember that his friends expressed their surprise that so much importance should be attached to so little apparent illness. The patient's pulse was somewhat slower than usual, and sometimes he was heavy and oppressed.

"I ventured to state that if we should not succeed in making the kidneys act, the patient would soon become comatose, and would probably die the following night; for this was the course of the malady in every other instance which I had seen. It happened so: he died in thirty hours after this, in a state of stupefaction.

"All the patients who have fallen under my care in this disease, were fat, corpulent men, between fifty and sixty years of age; and in three of them there was observed a remarkably strong urinous smell in the perspiration twenty-four hours before death. Only one of them had complained of previous nephritic ailment. He had suffered frequently, and had passed several small calculi: but there was no difference in the progress of his symptoms when the paralysis had once taken place.

"If any water, however small the quantity, had been made in these cases, I should have thought it possible that the patients might have recovered; for it has often surprised me to observe how small has been the measure of that excrementitious fluid which the frame has sometimes thrown off, and yet preserved itself harmless; but the cessation of the excretion altogether, is universally a fatal symptom in my experience, being followed by oppression on the brain." (P. 31.)

"*Tic Douloureux*," the pathology of which forms the subject of the third dissertation, Sir Henry considers, in its severest form, to be "connected with some preternatural growth of bone, or a deposition of bone in a part of the animal economy where it is not usually found, in a sound and healthy condition of it, or with a diseased bone."

In confirmation of this view, several cases are related, the chief of which occurred in the author's practice; others were communicated to him by competent authorities.

"A lady, forty years of age, suffered under the violent form of tic douloureux, at Brighton, notwithstanding the careful attention and skill of a very judicious physician there. On returning to town it was observed that the rending spasms, by which the disease is marked, were frequently preceded by an uneasiness in one particular tooth, which exhibited, however, no signs of unsoundness; but the constancy of this symptom was enough to justify the extraction of the tooth in this instance (though the failure of this expedient to afford relief in general does not encourage recourse to the operation), and, on its being drawn, a large exostosis was observed at the root of the tooth; and the lady never suffered more than very slight attacks, and those very seldom afterwards.

"The Duke of G. was attended by Dr. Baillie and myself for six weeks, under this disease, in its most marked and painful form, without deriving benefit from our prescriptions. At length we thought it best to advise him to repair to the sea-coast, in hopes of renovating his shattered system by taking bark there. After he had sojourned a month by the sea-side, a portion of bone exfoliated from the antrum Highmorianum, and the Duke recovered immediately, and has never suffered the disease since. The bone had been hurt probably by a fall from his horse which the Duke had met with some months before.

"'The late Earl of C. underwent martyrdom by this disease, and excited the warmest sympathy of his friends by the agonies he sustained for many years. He submitted to the operation for the division of several branches of the fifth pair of nerves repeatedly, by Sir Edward Home and by Mr. Charles Bell, without obtaining more than mere temporary relief. At length he was seized by apoplexy, and lay insensible for some days, and in great peril from the attack, but finally recovered. After the apoplexy, the paroxysms of the tic douloureux became less frequent and less severe, and were administered to satisfactorily by an ingenious physician, who wrote his inaugural exercise on the disease. For the last year or two of his life his Lordship had ceased to suffer from the tic, and died at an advanced age without any marked malady. His head was not examined after death, and therefore we are left to conjecture only what might have been the immediate cause of his former sufferings. Whilst I attended him, he underwent repeated exfoliations of the alveolar processes of the teeth, which I thought occasioned his torment; and to account for the cessation of the complaint, I supposed that these efforts to throw off diseased portions of bone might have ceased, or that the apoplexy had disqualified the nerves for suffering so exquisitely; but there might have been besides, as some later instances have made probable, disease in the bones of the head.

"The late Dr. P. fell a sacrifice to this dreadful disease, after sustaining its tortures, for some years, with a constancy which attracted all our pity and esteem, and died at last under apoplexy.

"No assistance which the experience of any of us could afford him, gave him relief or controlled the violence of the attacks. On examining his head after death, there was found an unusual thickness of the os frontis, where it had been sawn through above the frontal sinuses, and at its juncture with the parietal bones. There was discovered also in the falciform process of the dura mater, at a little distance from the crista galli, a small osseous substance, about three eighths of an inch in length, rather less in breadth, and about a line in thickness. The vessels of the pia mater were turgid with blood, and about an ounce of fluid occupied the ventricles. I lamented that the frontal sinuses had not been examined, for I remember he replied to a question which I once put to him, as to his ever having experienced any suppuration within any bony cavity, that he had twice suffered suppuration in the frontal sinuses.

"Dr. P. had submitted with great patience to a division of several branches of the fifth pair of nerves, under the judicious operation of Sir Astly Cooper, who, on my mentioning to him the notion I entertained of the tic douloureux, was so obliging as to shew me the skull of a person who had died of this disease in the country. The internal surface of the frontal bone is a perfect rockwork.

"All the cases which I have described have fallen under my own immediate observation. I will now add another, with which I have been favored by a fellow of the College, a physician of high character and eminence in one of the most populous towns in this island. It serves remarkably well to confirm the opinions I have thrown out in this paper. The unhappy sufferer was a lady advanced in life: at the age of sixty-five she was attacked with exquisite pain in the branches of the fifth pair of nerves, on the right cheek, nose, and temple, the tortures of which, and the dreadful 'clawings and scratchings,' to use her own words, were said to surpass all that was ever witnessed, and to set at nought all powers of description. For nearly ten years the paroxysms continued to recur with more or less of intermission. The operation of dividing the supra-orbital branch of the nerve was succeeded by an alleviation of pain during the following five months. Various plans of treatment were adopted, and it would be difficult to name any remedy which the patient did not try. Those which satisfied her most were carbonate of iron and valerian; of the former of which she took, in the course of her illness, twenty-seven pounds, and even more than that of the valerian. Opiates gave relief at night, but failed in the largest doses in the daytime. Her intellect was not impaired, nor was there any derangement of her general health, until after a time a most distressing dyspnœa occurred, with other symptoms of visceral disorder. She was free from pain during the last six months of her life, which was terminated at length by apoplexy. The head was opened after death, and an enormous thickening was observed of the frontal, ethmoidal, and sphenoidal bones, in one part to the extent of half an inch; and the anterior lobes of the brain were curiously moulded and indented by the thickened bone. There was thickening also of the whole of the cranium, but not to so great a degree any where as in the parts which have just been named." (P. 39.)

This paper concludes with some observations on sympathy, as producing or favoring the production of disease, such as chorea and epilepsy, dependent on disordered secretions, intestinal worms, &c., and on idiosyncracies, as where epileptic fits and strangury have followed the administration of rhubarb, not only in one individual, but where, as in the latter, the peculiarity was common to a whole family; many of which like instances must be familiar to every one. These are subjects of a very seducing kind for comment, but we restrain ourselves, lest we should be detected writing an essay instead of a review: indeed, our notice has already run to such a length, that we cannot do more than pass a general encomium on the remaining papers, and recommend them warmly to the perusal of our friends.

We did intend to have made some comments on, and had marked several passages for extraction, from the Harveian and Dedicatory Orations, many parts of both being peculiarly elegant in their style. The latter, on the whole, we rather like the best; but there are some parts of the former which are quite Ciceronian: vide page 124, "At neque in sylvis," &c.; page 131, "Aut quid collaudem," &c.; and same page, "Valeas, itaque, fortunate Senex!" &c., which is truly beautiful. The conclusion of this oration is also much finer than that of its successor, which we think sounds rather poor: it would have read better had "delabetur" been made the final word. The Latin is certainly the fittest language for uttering praise: it allows many things to be said, and in a higher tone, than our own more sober, perhaps more modest, tongue would consider justifiable. The giver of a library, even were it as extensive as the Alexandrian, might almost think himself requited by the paragraph beginning "At quanti Rex bonus," &c., page 145, and "Te igitur augustissime Rex!" &c., page 146. The introduction of BAILLIE'S

name is particularly happy, and the terms in which the praise is couched worthy both of the orator and of the man he lauds.

From the account of the appearances of the coffin and body of King Charles I., we should probably have made some extracts, had not our hebdomadal and diurnal contemporaries rendered the subject too common for our pages: we therefore close this little (would it were larger) volume; but we quit it with regret; and, hoping that Sir Henry's occupations will allow him, before long, to favor us with more of his Essays and Orations, we will only, in conclusion, add, that the present do honour both to his head and heart.

---

*On Canine Madness; comprising the Symptoms, Post-mortem Appearances, Nature, Origin, and Preventive and Curative Treatment, of Rabies in the Dog, and other Domestic Animals: being a Series of Papers published in "The Veterinarian," in* 1828, 1829, *and* 1830. By W. Youatt, v.s. and f.z.s., Lecturer on the Anatomy and Diseases of Domestic Animals, &c.—Longman, London.

We have, in another part of our Journal, stated, in general terms, the value of this little essay, which first appeared as a series of papers in "The Veterinarian." Mr. Youatt is well known to have had considerable experience of the dreadful malady upon which he writes; and he particularly claims our attention, because his main object is to state unvarnished facts, and not to support any abstract hypothesis. If it should be objected, that medical practitioners are but little likely to derive advantage from a history of rabies as it occurs in the dog and other animals, we answer, that it is from such a source we have most reason to expect our knowledge of the nature, and even treatment, of the disease in man, to be ultimately increased, if not perfected: and, besides, it is frequently expected that the surgeon should be prepared to determine whether a dog, or other animal, is or is not mad, in order that he may judge of the safety or danger of the person who may have been bitten or injured. It is essentially necessary, therefore, that he should be acquainted with the disease as it exists in animals, in order that he may be prepared to relieve unnecessary alarm, or to adopt a prompt and decisive practice.

Mr. Youatt first describes the symptoms of rabies in the dog; and here it will be at once perceived that, unlike some writers upon the same subject, he writes from that which he has seen, and not from what he has borrowed at second or third hand from the statements of others.

"The earliest symptoms of madness in the dog are sullenness; fidgetiness; continued shifting of posture; a stedfast gaze, expressing suspicion; but when directed on the master, soon clearing up, and followed by some action indicating affection.

"An earnest licking of some part, on which a scar is generally found. If the ear be the affected part, the dog is incessantly and violently scratching it. If it be the foot, he gnaws it until the integument is destroyed. He gets into a passion with it, and growls over it; and is so insensible to pain, that in one case the foot was dreadfully mangled, and in another the greater part of the penis was gnawed away.

"Considerable costiveness, occasional vomiting, and a depraved appetite, are very early found; bits of thread, hair, straw, dung, are picked up; and frequently the dog will lap his own urine, and devour his own excrement. The animal next becomes irritable; flies fiercely at strangers; mumbles the hand or foot of his master; is impatient of correction; seizes the stick of a whip; quarrels with his own companions; eagerly hunts out and worries the cats; demolishes his bed or carpet; gnaws and shakes his chain; makes the most violent efforts to escape; tears to pieces his kennel or the door by which he is confined, and sometimes breaks his tushes in the attempt. If he escapes, he usually attacks those dogs only that fall in his own way; or, if naturally ferocious, he will diligently and perseveringly seek his prey. He will overcome every obstacle to effect his purpose; and at length returns to his home completely exhausted.

"The desire to do mischief depends much on his previous disposition: it often proceeds not beyond an occasional snap, and then only when purposely irritated: but with the fighting dog, the scene is terrific; he springs to the end of his chain; he darts with ferocity at some object which he conceives to be within his reach; and is eagerly employed in destroying every thing around him.

"Very early in the disease the expression of the countenance is changed. The conjunctiva is occasionally highly injected, at other times scarcely affected; but the eyes have a peculiarly bright and dazzling appearance, accompanied by a slight strabismus; not the protrusion of the membrana nictitans, as in distemper, but an actual distortion from the natural axis of the eye: the lids of one eye are frequently contracted; twitchings begin around that eye, and gradually spread over the face.

"About the second day, a considerable discharge of saliva commences; but this does not continue more than ten or twelve hours, and is succeeded by an insatiable thirst: the dog is incessantly drinking, or attempting to drink; he plunges his muzzle into the water. When the flow of saliva has ceased, he appears to be annoyed by some viscid matter in the fauces, and, in the most eager and extraordinary manner, works with his paws at the corner of his mouth to get rid of it; and, while thus employed, frequently loses his balance, and rolls over.

"A loss of power over the voluntary muscles is now observed: it begins with the lower jaw, which hangs down, and the mouth is partially open, but by a sudden effort the dog can sometimes close it, although occasionally the paralysis is complete. The tongue is affected in a less degree: it protrudes from the mouth, and becomes of a leaden colour. The dog is able, however, to use it in the act of lapping, but the mouth is not sufficiently closed to retain the water: therefore, while he hangs over the fluid, eagerly lapping for several minutes, it is very little or not at all diminished. He catches at his food with an eager and ill-directed snap, and often fails in his attempt to seize it: he bolts it unchewed, or drops it in the act of chewing. In the more advanced stage of the disease, this paralysis frequently disappears from the head, and attacks the loins and extremities. A peculiar indecision attends every motion; the animal staggers about, and frequently falls: previous to this he is in almost incessant action; he scrapes his bed together, and disposes it in various forms; he starts up, and eagerly gazes at some real or imaginary object; he traces the fancied path of something floating around him; he fixes his eye intently on some spot on the wall or partition; he suddenly plunges at it; his eyes then close and his head droops; in an instant he starts and gazes wildly around. The voice of his master recalls him from this delirium; he acknowledges, and endeavours to fondle on him, but in a moment he is wandering again.

"He frequently, with his head erect, utters a short and very peculiar howl; or, if he barks, it is a hoarse inward sound, altogether dissimilar from his usual tone, and generally terminating with this characteristic howl. Respiration is always affected; often the breathing is very laborious, and the *inspiration* is attended with a very singular, grating, choking noise. On the fourth, fifth, or sixth day of the disease, he dies; occasionally in slight convulsions, but oftener without a struggle.

"Of the symptoms popularly but erroneously supposed to accompany rabies, I will only say, that the rabid dog never has fits: the existence of epilepsy is a clear proof that there is no rabies. There is *no dread of water; no spasm attending the effort to swallow;* but a most extraordinary and unquenchable thirst. There is *no fear excited in other dogs; no wondrous instinct warning them of danger.* There is *no peculiar and offensive smell; no running with the tail between the legs*, except when, weary and exhausted, he is seeking his home; *no pustules in or near the frænum of the tongue.*" (P. 1.)

Mr. Youatt states that the small glands under the membrane of the mouth are tumefied, and he suspects that an occasional enlargement of these has been mistaken for pustules.

A brief yet interesting sketch is given of the post-mortem appearances in the dog; and, in the next paper, the symptoms of rabies in the horse, the ox, the sheep, the swine, the cat, and the human being, are described.

"The symptoms of rabies are very similar in man, and in all our domesticated quadrupeds. In all there is the same affection of the respiratory nerves; the same howling, or at least choking noise; the same excessive excitability, and incessant and uncertain action; the same singular delirium, affection of the stomach, and discharge of saliva; the same inevitably fatal termination of the disease; and, I am disposed to believe, nearly the same morbid appearances after death.

"The human being, however, has a dread of water, which the quadruped has not. It is true that the dog is unable to swallow, but he flies eagerly to the water; and all other quadrupeds, with perhaps an occasional exception in the horse, drink with ease and with increased avidity.

"How is this? Are they different diseases? Is hydrophobia in the human being the creature of imagination?" (P. 11.)

Mr. Youatt proceeds to discuss these questions, by first pointing out the decided difference which exists in the symptoms and progress of various diseases, as they occur in man and animals.

"Hydrophobia, the dread of water, and the horrible spasms which accompany the attempt to swallow any fluid, depend on the highly irritable state of the fauces and larynx.

"The larynx is the guard of the trachea during the act of deglutition. The epiglottis is then pressed down upon the orifice of the larynx, and the crico-arytenoïdei muscles contract, and accurately close the aperture.

"The larynx likewise discharges a more important function. It is the principal organ of voice. According to the number of vibrations performed by the chordæ vocales in the act of expiration, is the precise tone uttered. A certain number of vibrations will produce a certain note. If the vibrations are quickened, the note assumes a higher place on the musical scale. If the number is lessened, the tone is proportionably graver or lower. The human voice is capable of nearly 500 different intonations, and each is produced by a determinate number of vibrations; and these vibrations, the number continuing the same, are modified, partly by the mechanism of the fauces and mouth, but more by the larynx, to express every varying emotion and passion. The larynx, then, must be possessed of exquisite sensibility, to obey so rapidly, and with such strict and almost inconceivable accuracy, the mandates of the will.

"The voice of quadrupeds is more confined. There are fewer differences of intonation, and the expression of passion is more simple. This exquisite sensibility will not be needed in them. It is a law of nature, that where extraordinary function is required, extraordinary sensibility and power are bestowed; but if the function is simple, and the work easily accomplished, less feeling and less energy are supplied. This is benevolent and wise. Quadrupeds have also a mechanism peculiar to each, and in a great measure independent of the glottis. The horse has a falciform membrane, attached by its middle to the thyroid cartilage, and its extremities extending to the margin of the rima glottidis. The ass has a similar apparatus, with an excavation under the thyroid cartilage, and two membranous sacs. The cat has a delicate membrane under the ligament of the glottis. The swine has membranous bags of a considerable size; and the ventriculi laryngis of the dog are very large.

"With this additional mechanism for the production of the voice, the extreme sensibility of the human larynx is not required; and nature bestows not that which the necessity of the animal does not demand.

"Considerable affection, however, of these parts attends every case of rabies in the dog. The involuntary and peculiar howl, the choking noise attendant on each respiration, and the blush of inflammation, more or less intense, which every dissection presents, sufficiently evince it.

"If we do not observe the dreadful spasms by which the human being is tortured, let it be remembered that these spasms are rarely excited by the passage of solid food. Fluids alone have this power, from their being brought into more intimate contact with the inflamed and irritable surface.

"It can easily be imagined, that the decreased sensibility which accompanies more limited and simple function would render those membranes not only indifferent to the passage of solids, but unaffected even by fluids.

"The mystery, therefore, is, in some degree, unravelled; and we not only cease to be surprised that rabies should be characterized by hydrophobia in the human subject, while there is no dread of water in the brute; but a consideration of the structure and functions of the larynx in man and the inferior animals, leads us to expect that something like this will occur." (P. 12.)

In the next paper, Mr. Y. endeavours to prove that rabies is an affection of the respiratory system of nerves; that it is caused by inoculation alone; that the virus must be received on some abraded, or wounded, or mucous surface; that it resides in the saliva alone, and that the power of the virus dies with the animal. The symptoms which have been described clearly indicate that rabies is a nervous affection, and particularly an affection of the respiratory system of nerves, or those which are employed in the instinctive and involuntary actions connected with respiration, and which serve to associate many of the voluntary muscles in the discharge of the same function.

"*Hydrophobia* has been produced in the human subject by the power of the imagination, or by strong excitement, but the disease has materially differed from rabies in its symptoms, progress, and termination." We have often seen hysterical women, and highly excited men, labour under *hydrophobia* in the proper, although not popular, acceptation of the term.

Mr. Youatt denies that rabies is ever spontaneous in the dog and his varieties: "in nineteen cases out of twenty, the inoculation can be proved, and in almost every case the possibility of it cannot be denied." Professor Trolliet, who has had extensive experience upon the subject, is opposed to our author upon this point: he has no doubt of the occasional spontaneous origin of rabies, but he limits its appearance in this form to the dog, the wolf, the fox, and the cat: all other animals, he believes, receive it from inoculation alone. Dr. Mason Good says that we have various examples on record, and that we have recently been furnished with another by Mr. Gillman, in which a dog, chained up in a yard, and cut off from all medium of contamination by other animals, has occasionally been attacked with genuine rabies, and exhibited its most decisive characters.

"The rabid virus must be received on some abraded or wounded, or mucous surface. On the sound integument it is harmless. There are a thousand proofs of this. Almost every author acknowledges it; and the writer of this essay has, with perfect impunity, had his hands many times covered with the saliva of the mad dog.

"I have said that the virus must be received on an abraded or mucous surface. The case, however, is not so clear with regard to the latter. A father, in the last stage of rabies, imprinted a parting kiss on the lips of his child; the infant died hydrophobous. A maiden would not be parted from her lover, and lavished on him her caresses, and escaped. The lips of the child might be chapped, those of the young woman might be unbroken.

"A man endeavoured to untie with his teeth a knot that had been firmly drawn in a cord: eight weeks afterwards he perished, undeniably rabid: it was then recollected, that with this cord a mad dog had been confined. A woman was attacked by a rabid dog, and escaped with the laceration of her gown. In the act of mending it, she thoughtlessly pressed down the seam with her teeth: she died. A physician, attached to a faithful spaniel on which symptoms of rabies were too apparent, ordered it to be destroyed; but, with pardonable weakness, he first kissed the poor animal. He paid the penalty of his imprudence with his life." (P. 19.)

Mr. Youatt confesses that the fact of the transference of the disease from the virus coming in contact with an unabraded mucous surface, cannot be perfectly decided. It is possible that the lips of those who perished might have been abraded; but he is strongly inclined to believe that the virus cannot be received on a mucous surface without imminent danger.

Does the rabid virus retain its energy after the death of the animal? Medical men, it is said, have taken it for granted that the disease might be communicated by the saliva, with equal certainty, before and after the death of the dog. We believe that this opinion is not general; the question has not been considered determined, and we are happy to find that Mr. Youatt intends to take every opportunity of settling it by the test of repeated experiment. His present belief is, "that the power of the virus ceases with the life of the animal." He has hitherto escaped, although, in many dissections, the saliva, notwithstanding considerable care, must have come into abundant contact with his hands, and they were not always sound.

The virus of every rabid animal may communicate the disease: it is chiefly propagated by the dog, because the teeth are his natural weapons of offence, and, when labouring under this malady, he has a strong disposition to bite. "The saliva of the cat, the fox, the badger, the wolf, the horse, the pig, the human being, have undoubtedly propagated rabies; and some say it has been propagated by the hen and the duck."

"Veterinarians should therefore be careful how they administer medicine, where there is the slightest reason to suspect the existence of rabies. If the hands be not perfectly sound (and of that we cannot be always sure) the mouth of the animal should be carefully avoided. Many farriers have perished from ignorantly or foolhardily drenching or balling a rabid dog; and a groom, not long ago, became hydrophobous from a scratch which he received in administering a ball to a horse." (P. 22.)

The virus does not appear to have the same effect on all animals. Two dogs out of three, bitten by one that is rabid, become rabid; the majority of horses inoculated with the virus perish. Cattle have more chance: the skin is looser, and less easily penetrated. With sheep the bite is even less dangerous, and probably because the tooth has been cleaned in its passage through the wool. The human being is least of all in danger, and JOHN HUNTER supposed that, if twenty persons were bitten, probably not more than one would become hydrophobic. "There can be no doubt," says Mr. Youatt, "that the decided majority escape, even if no means, or those which are inert and insufficient, are adopted: hence the falsely acquired reputation of so many prophylactics."

The wound inflicted by a rabid animal heals, as would another wound, according to its situation, magnitude, laceration, and the constitution of the patient. "Days, weeks, and months pass, and not the slightest circumstance occurs to indicate impending danger." After remaining dormant for an uncertain period, the virus commences its work of destruction: "the cicatrix generally begins to itch, and inflammation spreads around it. The diligent licking of some part where the mark of a bite can be traced, is an early and frequent symptom of rabies in the dog." The average period which elapses between the infliction of the bite and the occurrence of the disease has been variously stated, both with respect to men and animals. Mr. Youatt has never seen a case in which the disease occurred in less than seventeen days after the bite, and he would calculate the average time at five or six weeks: after three months he would consider the animal tolerably safe, but he has met with two instances, in one of which it occurred so late as the fifth, and in the other after the expiration of the seventh month, and where reinoculation was not suspected, and was scarcely possible.

We now approach the most important part of the subject, the preventive treatment, the consideration of which Mr. Y. premises by briefly relating a case of rabies in the dog, upon which Mr. Mayo made some experiments. The animal was labouring under well-marked symptoms of the disease. Seven ounces of blood were taken from the jugular vein, and an attempt was made to inject an equal quantity of warm water: the experiment did not perfectly succeed. Five or six ounces more blood were lost, and eight or ten ounces of water injected. Nothing more was done for a quarter of an hour. The laborious breathing and choking inspiration evidently increasing, the anterior portion of the second ring of the trachea was excised: "this afforded not the slightest relief; the hoarse, grating, choking inspiration was decidedly aggravated."

Mr. Youatt offers no decided objection to the application of a ligature above the bitten part, yet he places but little confidence in the utility of this measure, because "the virus lies in the wound inert until it has assimilated to itself other matter, and increased in quantity, or, by its continued presence, irritated the neighbouring parts, and disposed them to become affected." But as in some cases, both in men and animals, the virus has very speedily affected the constitution, and quickly destroyed the patient, we should certainly very strongly advise the use of a tight ligature above the injured part; as Dr. Mason Good observes, analogy is altogether in favor of this proceeding. It is well known to be one of the most important steps we can take in confining the poisonous effects of the rattlesnake, and other venomous animals.

"Excision of the part is the mode of prevention generally adopted by the human surgeon; and it would seem to be most judicious practice. If the virus is not received into the circulation, but lies dormant in the wound for a considerable period, the disease cannot supervene if the inoculated part be destroyed.

"Excision of the part has however frequently failed. Not a year passes without many lamentable instances of it. It has occurred in the practice of the most eminent surgeon; and it seems scarcely, or not at all, to impeach the skill of the operator.

"How do we account for this? The knife may penetrate the deep and tortuous recess of the wound, in which the virus is lodged, and then its track will be empoisoned. Or if the incision be freely made round the wound, and does not penetrate into it, the blood will follow the knife; a portion of it will enter into the wound inflicted by the dog; it will come in contact with the virus; it will be contaminated; it will overflow that cavity; it will be received into the new incision, and it will carry with it the seeds of disease and death.

"Aware of this, many practitioners use the caustic after the knife. Every portion of the new wound is submitted to its influence. Has the question never occurred to them, if the caustic be necessary to give security to the operation of excision, might not the knife have been spared, and the caustic alone used?

"It will be imagined, then, that I am an advocate for the use of the caustic. Most certainly. But what caustic? Not a liquid one. Not one that speedily deliquesces. For, in the first place, it is unmanageable; and, what is a more important consideration, it may hold in solution, and not decompose the poison, and thus inoculate the whole of the wound.

"The caustic which I would with much confidence recommend is the nitrate of silver. It is perfectly manageable. Being sharpened to a point, it may be applied with certainty to every recess and sinuosity of the wound.

"The potash, and the nitric acid, will destroy the substances with which they come in contact; but the combination of the caustic and the animal fibre will be a soft or semi-fluid mass. In this the virus is suspended, and with this it lies, or may be precipitated upon the living fibre beneath. Then there is danger of re-inoculation; and it would seem that this fatal process is often accomplished.

"The eschar formed by the lunar caustic is hard, dry, and insoluble. If the whole of the wound has been exposed to its action, an insoluble compound of animal fibre and the metallic salt is produced, in which the virus is wrapped up, and from which it cannot be separated. In a short time the dead matter sloughs off and the virus is thrown off with it.

"Previous to applying the caustic, it will sometimes be necessary to enlarge the wound, that every part may be fairly got at; and I would without hesitation amputate, if I were not fully assured that I could get at every part. The eschar having sloughed off, it will always be prudent to apply the caustic a second time, but rather more slightly, in order to destroy any part that may not have received the full influence of the first operation, or that, by possibility, might have been inoculated during the operation.

"Does any chemical combination take place? Is the virus neutralised by its union with the caustic? I cannot demonstrate this, but I have much reason to believe that some effect of this kind is produced.

"It is painful to speak of one's self; but I may, perhaps, here be permitted to say, that I have been bitten four times by dogs decidedly rabid: at each time I freely applied the caustic to the wound, and I am living to the present day. I have operated on more than four hundred persons, all bitten by dogs, respecting the nature of whose disease there could be no question:* I have not lost a patient. One poor fellow died of fright, but not one became hydrophobous. To what can I so naturally attribute this, as to some chemical affinity between the nitrate and the virus, by which an insoluble and inert compound is formed?" (P. 32.)

The bitten portion having been removed by the knife, or destroyed by caustic, the mildest means should be adopted to heal the wound as speedily as possible. It is, of course, important to determine at how long a period after the patient has been bitten the knife or the caustic may be applied, with a fair prospect of success. "The sooner the better:" but Mr. Y. would apply the caustic with confidence at any period before the appearance of the disease. Seventeen of his patients had been bitten more than a week before the operation, two more than a fortnight, and the majority more than twenty-four hours.

"Until some effect has been produced on the nervous fibrils in contact with the virus, and that, or the influence of it, has been conveyed to the common sensorium, and thence propagated over the frame, and thus new relations have been established with other and distant parts, I should not hesitate to operate. At one of our hospitals, amputation was not long ago performed above the bitten part, yet the poor fellow died: the disease had been established before the operation, and that series of morbid action had commenced which could not be arrested. There are facts, however, on

* "I am bound to add, that one of the surgeons of St. George's Hospital told me that, since his connexion with that establishment, he and his colleagues had operated on more than as many thousands, bitten by dogs, (he could not say that all of them were rabid,) and he was not aware that one of them had been lost. This, at least, is most consolatory, whatever may become of my theory of the caustic."

record, which will fully exculpate the surgeon who advised or performed the operation. It is related in the Medico-Chirurgical Annals of Altenburg, (September 1821,) that two men were bitten by a rabid dog: one became hydrophobous, and died; the other had evident symptoms of hydrophobia a few days afterwards; a surgeon excised the bitten part, and the disease disappeared. After a period of six days, the symptoms returned: the wound was examined; considerable fungus was sprouting from its bottom: this was extirpated; the hydrophobic symptoms were again removed, and the man did well.

"Trolliet relates a similar case. Several persons were bitten by a rabid wolf, and some of them died. The cicatrix in the arm of one man became inflamed, and gave him much pain: the caustic was freely applied, and no hydrophobic symptoms appeared.

"It is only, however, in the early stage of the disease, that we should be justified in destroying the part and torturing the patient; and, if we proceeded to operation, that operation should be complete and severe, not only to eradicate all the virus, but by a new and violent irritation, to cause possibly a salutary metastasis." (P. 35.)

In the eighth essay the author mentions various medicines which have been tried, and some with apparent success, as preventive remedies, as the Box, Alisma Plantago, Belladonna, Scutellaria, &c.

The ninth and last paper, on the cure of rabies, may be quickly passed over. Mr. Youatt has "nothing satisfactory to offer" upon the subject: "not one instance of successful treatment of rabies has occurred." He has, indeed, been told, by a veterinary practitioner, "*almost*" at the head of the profession, that he has cured several cases of rabies; and he calls upon that gentleman "respectfully but firmly to favor the medical world with the history of these cases." This appeal is very proper, but we conceive the "respectful" part of it might have been omitted: he who believes he possesses a remedy for rabies, and does not disclose it, is not entitled to any such courteous terms. To the long list of remedies which have from time to time been tried, the Guaco has recently been added, and somewhat too lavishly and inconsiderately praised: as yet, however, we have no satisfactory evidence of its curative power; but it is proper to mention that it was given to a hydrophobic patient in St. Thomas's Hospital, and that he experienced slight and temporary relief after every dose.

We have now given the substance of Mr. Youatt's excellent little work, and we cannot but express our gratitude to him for the very concise and perspicuous manner in which he has communicated the result of his extensive experience, upon a subject in which all medical practitioners must feel so deeply interested. This is the first time we have had the pleasure of noticing any work from a member of the veterinary profession: we trust it will not be the last, and that the ability and zeal of Mr. Youatt, which he has displayed upon this and many other occasions, will stimulate his brethren to give the world additional proofs of the rapidly improving state of veterinary medicine and veterinary practitioners.

---

*Observations on Distortions of the Spine; with a few Remarks on Deformities of the Legs.* By LIONEL J. BEALE, Member of the College of Surgeons, and Author of a "Treatise on Deformities."—8vo. pp. 102. Wilson, London.

SINCE the publication of Mr. BEALE's Treatise on Deformities, reviewed in our Journal for October 1830, p. 242, further observation has induced him to modify some of the opinions therein contained, and fresh facts have given rise to other views of some of the varieties of spinal curvature. A new edition of Mr. B.'s former work is called for; but, from the desire of rendering it a complete book of reference on the subjects of which it treats, he very wisely delays bringing forward the second edition until he has been enabled to collect much additional and important matter for comment. The object of the present tract is to elicit from others criticisms and opinions on the points discussed, in order that the author's own opinions may be either strengthened or modified by the accumulated experience of many.

We shall pass over the subjects Mr. Beale first discusses, for the purpose of giving more of our attention and space to the chapter on "*Spinal Irritation*," a pathologic condition which cannot too much interest the practitioner, both on account of the numerous and ever-varying symptoms it produces, and the frequent difficulty which exists of distinguishing the morbid phenomena arising from it, from diseases with which it has no connexion. It may be well, however, first to enumerate the subjects of the first part of the essay: they are, lateral curvature of the spinal column; chronic inflammation of the cartilages and ligaments of the spine; the effects of attitude; want of exercise; and the effects of stays and tight lacing.

*Of Spinal Irritation.* Several able writers have recently attracted the attention of the profession to this subject, but we must claim for our intelligent and experienced correspondents, Dr. and Mr. D. GRIFFIN, the merit of having given the most extended and practical history of it. The numerous cases they have detailed in our Journal,* together with the very judicious remarks and arguments by which those cases are accompanied, cannot be perused without the deepest interest by all practitioners. Since we perused the essays of Dr. and Mr. Griffin, our own practice has afforded us several opportunities of verifying the opinions they so ably maintain, and of ascertaining the efficacy of the practice they recommend.

Mr. Beale observes, that the term "spinal irritation" has been given to a condition of the spinal marrow, or the nerves as they emerge from it, which is supposed (proved, we think,) to be the immediate cause of many neuralgic and hysterical disorders. Several writers on spinal distortions have described symptoms which they have referred to incipient curvature, exactly similar to those which have of late been ascribed to a peculiar condition of the spinal marrow, or the nerves at their junction with it. This condition has by some been designated spinal irritation, and by others subacute inflammation. The similarity of the symptoms in these cases to those which occur in the early stage of spinal distortions, has induced Mr. B. to introduce a few remarks on the subject, which he the more readily does "because it is at present involved in some doubt, and it is very desirable that the observations of those who refer nervous and hysteric disorders to the spinal brain may be verified, as we shall then have a means of remedying diseases which have ever been among the opprobria of medicine. Many of our leading medical authorities are very sceptical as to the connexion of these complaints with spinal irritation, and consider the relief which follows leeching and blistering the spine as coincidences rather than consequences."

It is very true that we have occasionally heard some doubts expressed respecting the connexion between irritation of the spinal cord and nervous and hysterical disorders; but when we have succeeded in the somewhat difficult task of removing old prejudices, and of fixing the mind of the sceptic upon this point, to the evidence afforded by a careful examination of the spine, and the good effects which arise from local bleedings and counter-irritation, we have never failed to remove his doubts, and to convince him of the truth of the general doctrine. Who the "many of our leading medical authorities" are that are opposed to the opinions referred to, we

* London Medical and Physical Journal, 1829 and 1830. The excellent series of papers to which we refer commenced in December 1829, and are continued through this Journal for 1830.

know not; and we are rather inclined to believe that Mr. Beale confers this honourable distinction upon those who have not yet examined the subject with sufficient attention, either from their want of opportunity or their want of industry.

Mr. Beale coincides in opinion with those who refer neuralgic disorders "to some mischief in the spine;" but at present he is disposed to think that this is, in many cases, the early condition of spinal curvature, arising from chronic inflammation and lymphatic infiltration of the cartilages and ligaments connecting the vertebræ. Having referred to the papers of Dr. Brown in the Glasgow Medical Journal, and of Mr. Whatton in the February number of the North of England Medical and Surgical Journal, the works of Dr. Addison and Messrs. Teale and Tate, and the essays of Dr. and Mr. D. Griffin before mentioned, Mr. Beale proceeds by stating, that "from the mass of evidence adduced by these and other practitioners, we cannot refuse to grant that there is at least some probability that many nervous disorders, and a peculiar condition of the spinal marrow or nerves, bear the relations of effect and cause; and as this peculiar condition generally yields to local bleeding and counter-irritation, it may sometimes depend on subacute inflammation.

"The symptoms attending this condition of the spinal marrow or nerves are very varied and anomalous, and differ much in extent and intensity. In slight cases, irregular shooting pains occur in the muscles in different parts of the body; occasional headaches, palpitation of the heart, loss of appetite, startings and tremblings, with general disability to exertion. In more severe cases, uneasiness becomes fixed, darting lancinating pains occur in the chest, abdomen, or limbs. These and other nervous symptoms may have existed for months without exciting suspicion of the real nature of the disorder; and various kinds of treatment have been adopted, often unnecessary, and generally inadequate to the removal of the complaint, the one symptom characteristic of this disease being overlooked, viz. tenderness on pressure in some part or parts of the spinal column.

"In slight cases pressure can be borne without any great suffering, in others the tenderness is so great that, in running the finger along the spine, the instant the irritable spot is arrived at, the patient starts, and a degree of anguish is occasioned so excruciating as to produce the most violent spasms, which either go off gradually in repeated faintings, or subside into periodical and less painful dartings along the nerves, running from the part through all their different ramifications.—*Whatton.*

"This disease occurs in every region of the spine; it exists sometimes in different parts at the same time, and occasionally in the whole column. When the upper cervical nerves are affected, the seat of pain is in the back part and sides of the head, the muscles of the face and neck are stiff, and there is difficulty in moving the jaw. When the seat of the irritation is the lower cervical region, we find darting pains and cramps in the course of the axillary and brachial nerves, in the upper and fore-arm, the muscles about the shoulder-joint and those of the upper and lateral parts of the chest: the breasts are sometimes hard and painful, there is a feeling of depression and lassitude, often accompanied by sighings, tremblings, and other of the symptoms usually called nervous.

"When the disease is seated in the upper dorsal region, painful shootings occur along the intercostal muscles, the edges of the ribs and sternum, and peculiarly under one of the breasts, which is most commonly the left. When the malady is in the lower dorsal region, there is pain around the abdomen and over the stomach, soreness and smarting along the ribs, a sense of constriction across the chest or the pit of the stomach; sometimes a difficulty of breathing, and a burning sensation over the sternum and at the point of the ensiform cartilage. In some cases there is a degree of atony in the abdominal muscles, producing difficulty in the expulsion of the urine and fæces, irregular pains over the whole abdomen, and occasionally partial paralysis of the integuments covering the lateral parts of the belly and thighs. (*Whatton.*) When the lumbar nerves are affected, there is aching pain in the loins, soreness of the muscles and skin of the generative organs and upper parts of the thighs, painful dartings along the crural nerves down to the ankles and feet; there is trembling, unsteadiness, and diminution of power in the whole of the lower limbs.

"When the irritation has extended to the ganglionic system, we have, in addition to the above symptoms, irregular and spasmodic actions of the involuntary muscles, and derangement of the functions of those organs which derive their nervous energy from the ganglia to which the irritation has been communicated. When this occurs with regard to the cervical ganglia, we have violent and stabbing headachs, painful throbbings of the carotid and temporal arteries, fixed and heavy pain at the base of the skull: from the lower cervical ganglia the mischief may extend to the cardiac nerves, and this may possibly be the cause of the violent palpitations, tremors, and alarms of nervous persons. This disease, when originating in the dorsal region, will extend to that part of the ganglionic system which gives off nerves to the organs of digestion and nutrition: the diaphragm, stomach, liver, and spleen, the large and small intestines, as their appriopriate ganglia are affected, become in their turn liable to derangement. When the irritation has extended to the stomachic plexus of nerves, there will be, among other symptoms, a painful depression at the pit of the stomach, tenderness on pressure, unpleasant distention, especially after eating, incomplete digestion, flatulence, acidity, &c. In these cases, the sympathy of the stomach with the brain will propagate the irritation to the latter organ, and will thus produce all the melancholy forebodings of hypochondriacism. It is not my purpose to enlarge on this subject, or I should be led into the interminable mazes of those extraordinary complaints which, under the titles of hypochondriasis, hysteria, spleen, the English malady, &c., have perplexed patients and doctors in all ages. My object has been to give a summary view of a modern opinion with regard to these complaints, which still requires the sanction of experience.

"We have enumerated many symptoms which appertain to the long catalogue of those which characterise what have been denominated nervous disorders, and if experience affirms the conclusion that these distressing and perplexing maladies always have their cause in the spine, this will not be the most unimportant of the modern improvements in the art of healing. That such a connexion does exist in many instances we have abundant facts to testify; numerous cases are now on record, of nervous and hysteric complaints being removed by local applications to particular parts of the spine. This class of diseases have hitherto been so perplexing, and a remedial means has so long been a desideratum, that the spine should never be neglected in such disorders. By careful observations, we shall be enabled to verify the truth of the foregoing descriptions, and determine how far we are warranted in referring nervous disorders to irritation or subacute inflammation of the spinal brain, or the communicating nerves. If we succeed only here and there in a case, we shall be rewarded for our trouble; for, although these complaints are too often disregarded, and made the subject of ridicule, although they are never attended with any danger to life, yet there is no class of maladies more distressing to the sufferers themselves." (P. 59.)

In illustration of his preceding observations upon the subject of spinal irritation, Mr. Beale narrates thirteen cases: we select the fourth and fifth as examples.

"Case IV. Miss B., aged nineteen, had been long subject to coughs; had been considered, during the winter of 1829-30, to be labouring under pneumonia and inflammation of the liver: her difficulty of breathing at times was so great, as to appear to threaten suffocation; her bowels were extremely torpid, and these attacks of supposed pneumonia and inflammation of the liver repeatedly recurred. The treatment to which she was subjected necessarily weakened her; and in the last summer she was removed to the coast, where she in some degree recovered her strength.

"On her return to town, she was extremely delicate and weak, her appetite capricious, her bowels inactive without medicine; she still had frequent attacks of pain in the chest, difficulty of breathing, cough and expectoration, and, in the eyes of her friends, she was considered consumptive.

"At this period, when I first saw her, she was extremely thin, her countenance sallow, her eyes dull, her tongue coated, respiration rather hurried, occasional dry cough, pulse weak and frequent; she complained of palpitation of the heart and fluttering at the pit of the stomach, some degree of tenderness over the region of the liver and stomach; the bowels seldom acting oftener than once in three days, and then not without medicine. Her sleep was unrefreshing, seldom having more than two or three hours during the night.

"I found, on inquiry, that before and during the paroxysm of difficulty of breathing, &c., which generally recurred once in a week or fortnight, she experienced a sense of choking, that her heart beat much, and that her urine was pale and in large quantity: from these circumstances, and her general appearance, I had no hesitation in concluding all her suffering to be neuralgic or hysterical; and, as the uterine functions were regular, I pursued my inquiries to the spine. On examination, a curvature of the dorsal vertebræ was discovered, the convexity towards the left side, the ribs on this side being preternaturally separated, while those of the right side were approximated. The left shoulderblade was nearer to the spine by an inch and a half than the right, but there was no material difference in their elevation. There was no pain on pressure, either on the spinous processes or at their sides.

"On extending the spine the curvature was much lessened which not only proved the recent state of the distortion, but the probability of an entire cure. The recumbent position, with occasional extension, was strictly adhered to for the first two months, after which exercises were employed, principally that of raising a weight over a pulley, by the action of the muscles of the neck and back. Mild cathartics and a regulated system of diet, after a time restored the action of the digestive organs. Tonics were employed to give tone to the system, and a few months restored this young lady to better health that she had experienced for some years.

"*Remarks.* This case exhibits an example of the sympathetic affections which sometimes result from pressure or irritation of the spinal marrow, or the nerves at their exit. We have neuralgic affections simulating the most important diseases. It is an instance of the necessity of a strict investigation before we determine the nature, and consequently the treatment, applicable to such cases. In delicate women, we should always hesitate before pronouncing a verdict of inflammation: it is hardly probable that such persons should be liable to violent inflammatory affections; and, as far as my experience goes, I should say that, in such subjects, what ought to be fairly denominated inflammation is rare; and I am quite certain that many delicate females have been sacrificed to the indiscriminate employment of anti-inflammatory remedies. There is a peculiar nervous mobility, call it what you will, in the female constitution, or something in their manner of life, which disposes them to neuralgic or congestive diseases, and but little to increased arterial action. The uterine system, from puberty, commences its influence, and there is no organ or function which is not occasionally influenced by sympathy with it. A delicate, pale-faced, bloodless female complains of pain in the side, may have a slight cough, her breathing is hurried, her pulse frequent, her appetite bad, her tongue coated: a routine practitioner pronounces it a case of pleurisy, the lancet is called into play, drastic purgatives are administered, foxglove or colchicum is prescribed, and that irritation which really constituted the complaint is kept up by the treatment adopted. Perhaps the symptoms are aggravated, the obstinacy of the disease calls for farther antiphlogistic remedies, and the patient dies, or falls into a state of apparent consumption.

"Such cases are not uncommon, and it behoves the young practitioner to endeavour to form a correct judgment with regard to them. Unfortunately, the kind of cases which are seen by students at hospitals and dispensaries, being chiefly those of the labouring classes, and for the most part inflammatory, misleads the judgment, and he commences practice with the admonitions of his teacher and his own experience, leading him to see inflammation at every step. It is often many years before the prejudices imbibed in the schools are destroyed by real experience of the diseases of a higher grade of society; but time at length convinces him that, as there is a nervous as well as a vascular system, so there are as many, and perhaps more nervous than vascular or inflammatory diseases.

"Case V. Miss ——, aged twenty-six, had long been in a deranged state of health, consulted me in April 1822: at this time she complained of pain in her back and side, her complexion was sallow, her person thin, and she bore the appearance of one who had suffered from long-continued illness. Upon inquiry, I found that, for several years, she had been subject to repeated attacks of what were considered inflammatory disorders, sometimes in the chest, but more frequently in the viscera of the abdomen: general and local bleedings always relieved her, and after these and other remedies she recovered, and usually enjoyed a fair degree of health for a few weeks or months, when fresh attacks occurred, and gave way to corresponding remedies. When I first saw her, she had slight fever; quick, small, and slightly intermitting pulse; her tongue was covered with a dirty yellowish fur; the stomach was irritable, and frequently rejected its contents; the bowels were not confined, but the motions had been very scanty for some time.

"The pain was in the left side, and was increased by pressure on the ribs: in fact, it was rather tenderness than pain, for the slightest touch was complained of. This is commonly enough the case in neuralgic pains, and, when it does occur, it leaves no room for mistaking it for the pain of inflammation.

"I found her lying on a sofa, having some time before consulted an eminent surgeon in a large provincial town, who was of opinion that there was a disease of the spine, and had enjoined an unremitting horizontal posture. This plan she had now adopted for some time, without benefit: on the contrary, her symptoms were much more severe than they had been. On examination of the vertebral column, I could perceive no point where there was the slightest deviation, nor was there any part of the spine painful on pressure: it is true she complained of the loins generally, but the pain was seated in the muscles, being increased more by motion than by pressure or percussion. I found, on inquiry, that the uterine functions were impaired, the catamenia being irregular in their periods, scanty in quantity, and unusually dark.

"I have no hesitation in determining this to be a case of hysteria, and that there was no disease whatever in the spine: how far such cases may depend on a disturbed state of the nervous power, imparted by the spinal marrow, I shall not pretend to say; but it does seem probable, from the fact that counter-irritation applied to that part of the spine from which the nerves supplying the seat of pain communicate. This has been strikingly illustrated by several modern writers on hysteria.

"To return to our patient. The local pain in the side was removed by a blister: she was ordered Pil. Hyd. and Ext. Hyosciam. of each five grains every night, and the Mist. Ferri c. twice in the day, with a purgative draught every second morning. Under this plan of treatment, the digestive functions improved; and, as all belief of spinal disease was dismissed, the recumbent position was abandoned, and she was permitted to take as much exercise as possible in the open air. After taking steel and occasional purgatives, the uterine functions performed their duty, and so long as she continued exercise in the open air her health improved, although I have since learnt she has had a repetition of her old attacks." (P. 69.)

The author concludes the work with a few brief remarks on distortions of the legs.

The present essay is a very valuable addition to Mr. Beale's previous Treatise on Deformities: he treats the subjects he has selected with great judgment, and we cannot too strongly recommend both his works to the attention of practitioners, and especially to medical students.

